# Continuous Community Engagement Is Needed to Improve Adherence to Ebola Response Activities and Survivorship During Ebola Outbreaks

**DOI:** 10.9745/GHSP-D-23-00006

**Published:** 2024-08-27

**Authors:** Gnakub Norbert Soke, Peter Fonjungo, Gisele Mbuyi, Richard Luce, John Klena, Mary Choi, John Kombe, Gerry Makaya, Francis Mbuyi, Henriette Bulambo, Mathias Mossoko, Celestin Mwanzembe, Bienvenu Ikomo, Pierre Adikey, Joel Montgomery, Trevor Shoemaker, Placide Mbala, Giulia Earle-Richardson, Dieudonne Mwamba, Jean-Jacques Muyembe Tamfum

**Affiliations:** a Division for Global Health Protection, Global Health Center, Centers for Disease Control and Prevention, Atlanta, GA, USA.; b Division for Global HIV and Tuberculosis, Global Health Center, Centers for Disease Control and Prevention, Atlanta, GA, USA.; c Directorate of Disease Control, Democratic Republic of the Congo Ministry of Health, Prevention and Hygiene, Kinshasa, Democratic Republic of the Congo.; d Viral Special Pathogens Branch, National Center for Emerging and Zoonotic Infectious Diseases, Centers for Disease Control and Prevention, Atlanta, GA, USA.; e Vysnova partners, Landover, MD, USA.; f Equateur Provincial Division of Health. Democratic Republic of the Congo Ministry of Health, Prevention and Hygiene, Kinshasa, Democratic Republic of the Congo.; g National Institute for Biomedical Research, Democratic Republic of the Congo Ministry of Health, Prevention and Hygiene, Kinshasa, Democratic Republic of the Congo.

## Abstract

Engaging communities in Ebola preparedness activities between Ebola outbreaks can not only improve community adherence to response interventions but also potentially help to improve survivorship in these communities during future Ebola outbreaks.

## Key Messages

- Despite improvements in care management with the development of a new Ebola vaccine and therapeutics, survivorship is still low (between 40% and 50%) in recent outbreaks due to low community acceptance of these lifesaving interventions.
- During an outbreak, the top-down strategy of control measures prioritizing case detection and management over risk communication and community engagement fails to address public concerns about the disease and available interventions, such as vaccines and therapeutics.
- When the outbreak is over, the intense level of outbreak response activities ends abruptly and activities that maintain community awareness and engagement rarely continue, fueling communities’ feelings of mistrust and abandonment.
- In high-risk communities for Ebola outbreaks, it is important that external partners work with the local communities to maintain Ebola preparedness activities between Ebola outbreaks.
- The continuous engagement with these communities will improve their acceptance of the existence of the disease and their adherence to response activities and interventions, such as vaccines and therapeutics, during the next Ebola outbreak. This better adherence can improve survivorship in these communities during the next Ebola outbreak.

## INTRODUCTION

In the Democratic Republic of the Congo (DRC), Ebola outbreaks have been more frequently reported in recent years, with a shortening of the intervals between outbreaks.^1^ Several factors have driven this change, including the impacts of deforestation on increasing small-scale cropland and mining, resulting in increased contact between humans and presumptive animal species suspected of Ebola transmission, such as fruit bats^2^^–^^3^; increasing numbers of Ebola survivors and the possible transmission through sexual intercourse,^4^ and improved detection of Ebola through better diagnostic testing.^5^ From August to October 2022, The Ministry of Health responded to the DRC’s 15th Ebola virus disease (EVD) outbreak since the first outbreak was reported in 1976, including 7 outbreaks during the last 4 years. Following the 2014–2016 West Africa and 2018–2020 Eastern DRC (10th) Ebola outbreaks, there have been significant scientific advances in the management of outbreaks caused by Zaire ebolavirus (EBOV), including the development and approval of an Ebola vaccine (Ervebo) and 2 specific monoclonal antibody treatments (Ebanga and Inmazeb).^2^^,^^3^ Initial studies demonstrated that these 2 therapeutic agents reduce EVD mortality caused by EBOV. Compared to the control group, mortality at 28 days was significantly reduced by 29% for patients who received Ebanga and by 35% for those who received Inmazeb. Further, late reception of treatment was associated with high mortality as the odds of death increased by 11% for each day that the patient was not admitted to the Ebola treatment center after the onset of symptoms.^6^ Despite these advances in EVD management, the case-fatality rates for EBOV outbreaks, which range from 70%–90% when untreated, remain high, averaging between 42% and 60% in recent outbreaks in the DRC (unpublished situational reports data for the 13th and 14th outbreaks).

Although these lifesaving interventions have been made available, members of affected communities have not always welcomed them.^7^^–^^9^ For example, during the 14th Ebola outbreak in Equateur in 2022, the uptake of the Ebola vaccine was low—with only 302 of the 1,076 contacts listed (28%) receiving the vaccine, and most vaccinated (1,307) were health care workers. Similarly, reluctance and delays in receiving Ebola treatment were also noted during the 14th EVD outbreak, resulting in only 25% of confirmed cases receiving EVD monoclonal therapy. While logistical issues (e.g., unavailability of cold chains) can impede the delivery of vaccines and therapeutics,^7^ it is unlikely that the low uptake of vaccination and therapeutics observed during the 14th EVD outbreak was due to logistical challenges alone. For example, Inungu et al. cite that while 4 investigational therapeutics were available to the Ministry of Health of DRC during the 2018 Eastern DRC Ebola outbreak, levels of general insecurity, distrust of government and foreign actors, and regional violence contributed to widespread community resistance to their acceptance.^8^ Furthermore, at the Fifth One Health Congress held in Canada in 2018, the lack of community trust and the lack of respect and understanding of specific community sociocultural needs were listed as impediments to the effective use of available therapeutics during the 2018 Eastern DRC Ebola outbreak.^9^ Anecdotal information reported during the 14th EVD response meetings suggests high levels of community reluctance to both the Ebola vaccine and acceptance for transfer to the Ebola treatment center to receive therapeutics (unpublished situational reports data).

To increase acceptance of Ebola vaccines and therapeutics, it is essential to understand why affected communities resist these lifesaving interventions, especially communities like those in Mbandaka (Equateur province) that have experienced repeated Ebola outbreaks in recent years. Analyses of data collected from community feedback during the 10th Ebola outbreak in Eastern DRC revealed the following themes around EVD treatment: uncertainty about whether effective treatment exists, mistrust of medical staff treating EVD cases (who were not their usual providers of care), and fear caused by the rapid deterioration of some patients with late-stage EVD admitted at the Ebola treatment center. There were 2 additional themes specific to vaccination: a perception that selectively vaccinating close contacts as part of the “ring vaccination strategy” was “discriminatory” (i.e., selection criteria were personal rather than medical) and a belief that not everyone was receiving vaccines with the same efficacy, possibly due to variability in the health outcomes of those who were vaccinated.^10^ During the 14th EBOV in the Equateur province, situational reports data revealed that a considerable proportion of the population still did not believe that Ebola exists and thought that this was a “manufactured outbreak” to benefit politicians and humanitarian organizations and to delay a planned visit of the DRC President to the Equateur province (unpublished situational report data).

## LACK OF COMMUNITY ENGAGEMENT HAMPERS EBOLA RESPONSE EFFORTS

The introduction of an intervention into a community is likely to be more readily accepted if community engagement and empowerment are sought before implementation. The cardinal principles of health care must be observed—even during an Ebola outbreak—which includes effective and transparent communication regarding risks and benefits.^11^ This goes beyond our usual biomedical approach of explaining risks and benefits. It is important that investigators put themselves “in the shoes” of community members and understand what will work best from their perspective. It is known from past outbreaks that communities collaborate better during EVD outbreaks when they have some meaningful inputs into the implementation of control measures.^12^ Unfortunately, during EVD outbreaks, most control measures are prescribed by disease experts and then given to communities for implementation, with little room for discussion of specific societal restrictions that might impede uptake. This top-down strategy is frequently rejected by the communities, which subsequently invest little effort in supporting implementation.^13^ It is important to acknowledge that during the early stages of any public emergency, when information collection and processing are imperfect, the ability to obtain public participation quickly is critical but not easily achieved, especially if part of the strategy is to discourage activities that are widely accepted in the community, such as social gatherings. Further, community-based activities do not receive the same level of attention and funding as other interventions during and after an EVD outbreak. In the active phase of an EVD response, activities that focus on case detection and case management are prioritized. Thus, surveillance, laboratory testing, infection prevention and control, and patient care are viewed as the “cornerstones” of the response. In contrast, risk communication and community engagement activities are considered secondary, even though such cornerstone activities cannot succeed without effective community engagement. A similar situation is observed during the post-Ebola enhanced surveillance period, where the emphasis is on surveillance and laboratory testing in survivors and the general population, while activities that maintain a level of community awareness and engagement are seldom funded.

Worse, this intense focus on Ebola response efforts rapidly dissipates when the number of cases declines. This may fuel cynical community perceptions about an “Ebola business,” in which response partner organizations benefit financially from the presence of an EVD outbreak in the affected area. As stated earlier, a considerable proportion of the population in the town of Mbandaka still considered the 14th EVD outbreak to be a “manufactured crisis,” even after this community had experienced 2 outbreaks in the past 3 years (unpublished situational reports data). Thus, in our view, it is not only the weak support of community engagement during the height of an outbreak but also the rapidity with which such efforts are abandoned that fuels mistrust that further impedes future EVD response efforts.

## CONCLUSION

In summary, it is critically important that during and after EVD outbreaks, the health behaviors and perspectives of affected communities be recognized, with possible concerns within the target community identified and addressed, and, just as surveillance and laboratory testing activities are prioritized, so too are community engagement and sensitization activities. New approaches to control EVD outbreaks should place local people at the center of all preparedness, response, and recovery activities.^14^ Involving the communities at every stage will help with ownership of these interventions and facilitate their uptake by the communities. For example, during the introduction of the Ebola vaccine in Guinea, champions from the community were identified to lead these campaigns and acted as role models to convince those from their communities to accept this new vaccine.^15^ Mechanisms for ongoing collection of community feedback can be established to regularly monitor preventive behaviors and public preparedness. Strategies must be put in place in high-risk provinces, such as North Kivu and Equateur in DRC, to maintain Ebola awareness among local communities. A more continuous focus on sociobehavioral aspects of the EVD response will build trust and confidence through community engagement and encourage adherence to public health interventions, like vaccines and therapeutics, thus improving survivorship during future EVD outbreaks.

## Notes

**Peer Reviewed**

**First Published Online:** July 29, 2024.
